# Survey of Activated FLT3 Signaling in Leukemia

**DOI:** 10.1371/journal.pone.0019169

**Published:** 2011-04-28

**Authors:** Ting-lei Gu, Julie Nardone, Yi Wang, Marc Loriaux, Judit Villén, Sean Beausoleil, Meghan Tucker, Jon Kornhauser, Jianmin Ren, Joan MacNeill, Steven P. Gygi, Brian J. Druker, Michael C. Heinrich, John Rush, Roberto D. Polakiewicz

**Affiliations:** 1 Cell Signaling Technology, Inc., Danvers, Massachusetts, United States of America; 2 Department of Pathology, Oregon Health & Science University, Portland, Oregon, United States of America; 3 Department of Cell Biology, Harvard Medical School, Boston, Massachusetts, United States of America; 4 Department of Hematology and Medical Oncology, Oregon Health & Science University, Portland, Oregon, United States of America; 5 Howard Hughes Medical Institute, Portland, Oregon, United States of America; 6 Portland VA Medical Center, Portland, Oregon, United States of America; Emory University, United States of America

## Abstract

Activating mutations of FMS-like tyrosine kinase-3 (FLT3) are found in approximately 30% of patients with acute myeloid leukemia (AML). FLT3 is therefore an attractive drug target. However, the molecular mechanisms by which FLT3 mutations lead to cell transformation in AML remain unclear. To develop a better understanding of FLT3 signaling as well as its downstream effectors, we performed detailed phosphoproteomic analysis of FLT3 signaling in human leukemia cells. We identified over 1000 tyrosine phosphorylation sites from about 750 proteins in both AML (wild type and mutant FLT3) and B cell acute lymphoblastic leukemia (normal and amplification of FLT3) cell lines. Furthermore, using stable isotope labeling by amino acids in cell culture (SILAC), we were able to quantified over 400 phosphorylation sites (pTyr, pSer, and pThr) that were responsive to FLT3 inhibition in FLT3 driven human leukemia cell lines. We also extended this phosphoproteomic analysis on bone marrow from primary AML patient samples, and identify over 200 tyrosine and 800 serine/threonine phosphorylation sites in vivo. This study showed that oncogenic FLT3 regulates proteins involving diverse cellular processes and affects multiple signaling pathways in human leukemia that we previously appreciated, such as Fc epsilon RI-mediated signaling, BCR, and CD40 signaling pathways. It provides a valuable resource for investigation of oncogenic FLT3 signaling in human leukemia.

## Introduction

FMS-like tyrosine kinase-3 (FLT3) is a class III receptor tyrosine kinase (RTK) that also includes C-KIT, C-FMS, and platelet-derived growth factor receptor (PDGFR). Type III RTKs share a common structure consisting of 5 extracellular immunoglobulin-like domains, a single transmembrane domain, a cytoplasmic juxtamembrane region, and a cytoplasmic kinase domain interrupted by the kinase insert. FLT3 is primarily expressed in early myeloid and lymphoid progenitors and plays an important role in their proliferation and differentiation. In human acute leukemia, FLT3 is expressed on the surface of the leukemic cells in 70–90% acute myeloid leukemia (AML) patients and most B-acute lymphoblastic leukemia (B-ALL) [1], [2], [3].

Overexpression and activating mutations of receptor tyrosine kinases (RTKs) are known to be involved in the pathogenesis of many types of cancer. Mutations in FLT3 are commonly detected in patients with AML. The most common of these mutations are internal tandem duplications (ITDs), which occur in 25–30% of these patients [4], [5], [6]. FLT3-ITDs are formed by duplication of a fragment in the juxtamembrane domain that is always in frame but varies in length. These mutations result in constitutive activation of FLT3. In addition, FLT3 has been implicated in the pathogenesis of infant and childhood ALL. Gene expression analyses have shown that FLT3 is highly expressed in *MLL*-rearranged acute lymphoblastic leukemias [7], [8], [9].

FLT3-ITDs activate many of the same signal transduction pathways as the native receptor, such as RAS-MAPK pathway, JAK-STAT pathway, PI3K, SRC, and phospholipase C-gamma [10]. FLT3-ITDs are associated with poor prognosis for AML patients [4], [11], [12] suggesting FLT3 or its downstream effectors as potential therapeutic targets. Efforts to target FLT3 have led to the development of several novel small molecule inhibitors. So far, the results from the initial trials were somewhat ambiguous [13], [14]. Thus, a better understanding of FLT3 signaling as well as its downstream mediators could provide new insights into the molecular mechanism and alternative drug targets for AML with FLT3-ITD mutation. Recently, two large-scale phosphoproteomics studies were performed from FLT3 mutant expressing BaF3 and 32D cell lines, and provide important insights into mutant FLT3 signaling in murine systems [15], [16]. Choudhary et al. identified 61 tyrosine phosphorylation sites that were upregulated by FLT3-ITD, and provided important information with regard to spatial regulation of FLT3-ITD signaling [15].

To gain a global view of oncogenic FLT3 signaling in human leukemia cells, we applied PhosphoScan®, an immunoaffinity-MS profiling approach to leukemia cell lines containing activated FLT3 mutations (FLT3-ITD and amplification) [17]. We identified many tyrosine phosphorylation sites unique to FLT3-ITD human AML cell lines, including a novel site from FLT3. To further investigate the FLT3 signaling pathway, we combined PhosphoScan®, with stable isotopic amino acids in cell culture (SILAC) to quantify changes in phosphorylation due to FLT3 inhibition in human leukemia cell lines. This experiment identified many phosphorylation sites regulated by FLT3. Finally, we identified novel protein phosphorylation sites from bone marrows of primary AML patients, and shed light on FLT3 signaling in vivo.

## Results

### Phosphotyrosine profiling of acute myeloid leukemia cell lines

**Figure 1:**
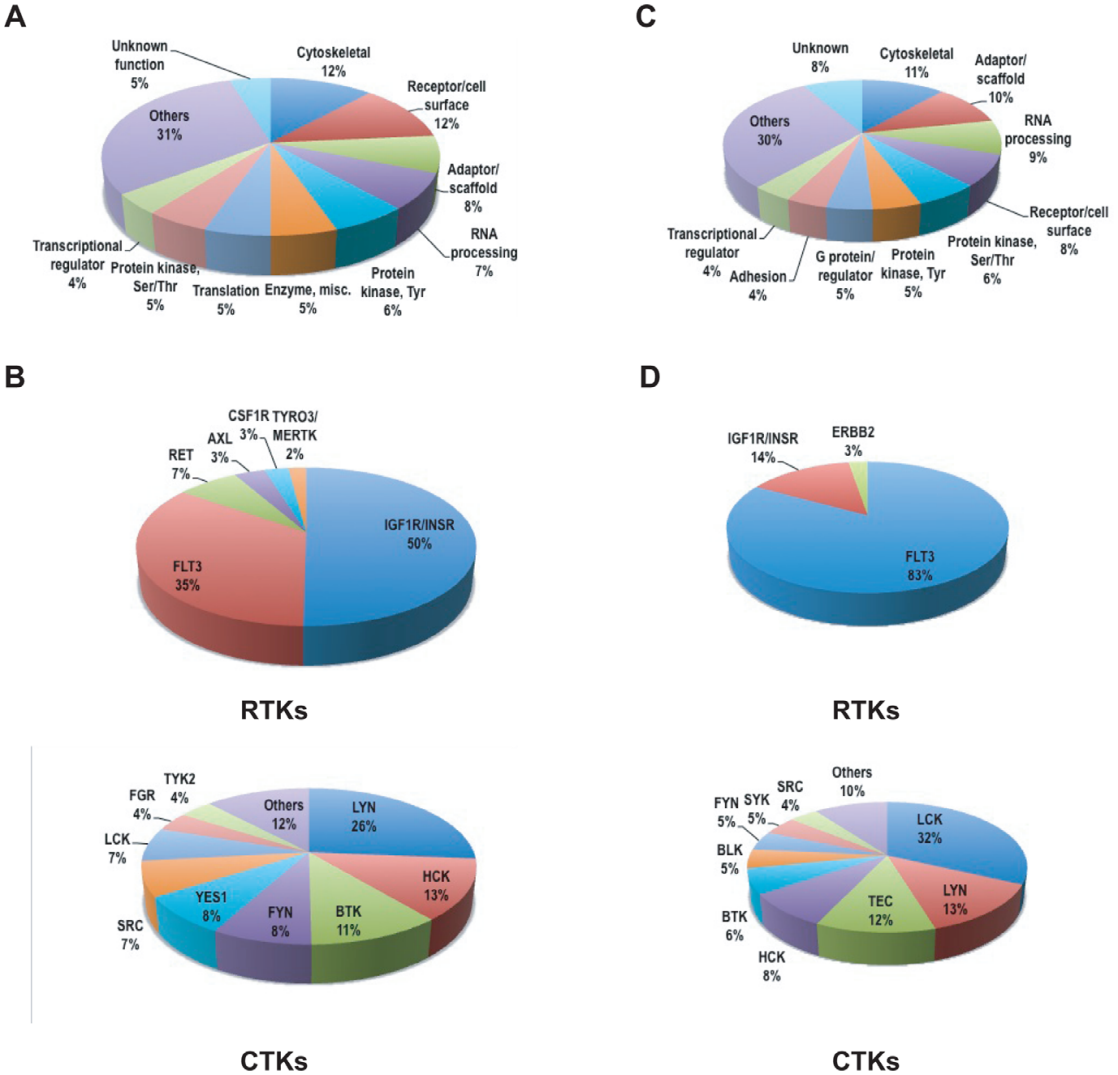
Phosphotyrosine profiling of AML and B-ALL cell lines. (A) Distribution of phosphoprotein types in AML cell lines. Each observed phosphoprotein was assigned a protein category from PhosphoSite ontology. The numbers of unique proteins in each category, as a fraction of the total, are presented. (B) Distribution of receptor tyrosine kinases (RTKs) and non-receptor tyrosine kinases (CTKs) in AML cell lines. The total number of spectral counts of each RTK/CTK is normalized against total number of phosphopeptides of GSK3A (100) in each sample, then the sum of the normalized number of each RTK/CTK as fractions of the total are shown. (C) Distribution of phosphoprotein types in B-ALL cell lines. Each observed phosphoprotein was assigned a protein category from PhosphoSite ontology. The numbers of unique proteins in each category, as a fraction of the total, are presented. (D) Distribution of receptor tyrosine kinases (RTKs) and non-receptor tyrosine kinases (CTKs) in B-ALL cell lines. The total number of spectral counts of each RTK/CTK is normalized against total number of phosphopeptides of GSK3A (100) in each sample, then the sum of the normalized number of each RTK/CTK as fractions of the total RTK spectra were represented.

Whole cell lysates from 8 AML cell lines were digested with trypsin, phosphopeptides were immunoprecipitated with phosphotyrosine antibody (pY-100), and analyzed by LC-MS/MS mass spectrometry (IAP-MS) [17]. Data from duplicate experiments were combined. Using this approach, we identified a total of 552 non-redundant phosphorylation sites in 513 proteins from 8 AML cell lines (Table S1 and S2). These data have been deposited in PhosphoSitePlus™ (www.phosphosite.org). Based on protein classification, cytoskeletal proteins, cell surface molecules, and adaptor and scaffold proteins are the most highly phosphorylated protein types (Figure 1A). Moreover, IGF1R and FLT3 are the most abundantly phosphorylated receptor tyrosine kinases (RTKs) in these cell lines (Figure 1B), consistent with their roles in the pathogenesis of AML [18]. However, IGF1R is preferentially phosphorylated in wild type FLT3 leukemia cell lines (Table S2). On the other hand, LYN, HCK and BTK make up the majority of cytoplasmic tyrosine kinases (CTKs) phosphorylation. In addition to the two known FLT3 tyrosine phosphorylation sites (Y842 and Y955) [19], we identified a novel FLT3 tyrosine phosphorylation site (Y630) in MV(4;11) (Table S2). Y630 is located in the first part of FLT3 split kinase domain. Its corresponding residue in the mouse is FLT3 is Y631. The substitution of tyrosine to phenylalanine at codon 631 did not impair the phosphorylation of murine mutant form of FLT3^Asp838Val^ [20]. However, the role of Y630 in the activation and signaling in the context of FLT3-ITD needs to be determined.

### Phosphotyrosine profiling of B-cell ALL cell lines

Since FLT3 is also involved in the pathogenesis of B cell acute lymphoblastic leukemia (B-ALL), we investigated FLT3 tyrosine signaling in three B-ALL cell lines, SEM, RS(4;11) and REH. While SEM cells express high level of FLT3, RS(4;11) and REH cells express relative low levels of wild type FLT3. In addition, SEM, but not RS(4;11) and REH, is sensitive to FLT3 inhibitor[8] (Figure S1A). Phosphotyrosine profiling identified a total of 533 non-redundant phosphorylation sites in 468 proteins from duplicate runs of these cell lines (Table S1 and S2). Similar to AML cell lines, cytoskeletal proteins, adaptor and scaffold, and proteins involved in RNA processing are the top three protein categories (Figure 1C). While FLT3 is the dominant RTK in these B-ALL cell lines, LCK, LYN, and TEC represent the major CTKs (Figure 1D), consistent with the role of these tyrosine kinases in antigen receptor signaling in lymphocytes. Of note, two additional FLT3 tyrosine phosphorylation sites (Y726 and Y969) were detected in SEM cells (Table S2). Besides tyrosine phosphorylation of FLT3 in SEM cells, we detected tyrosine phosphorylation of proteins involving B cell receptor (BCR) signaling, such as CD19, BCAP, LAB, BTK, LYN, GRB2, GAB1/2/3 [21], [22], [23], [24], [25], [26], [27], [28]. This observation raises the possibility that in B-ALL FLT3 takes advantage of the BCR signaling complex to promote B cell proliferation and survival.

### Identification of MLN518-responsive tyrosine phosphorylation sites

**Figure 2:**
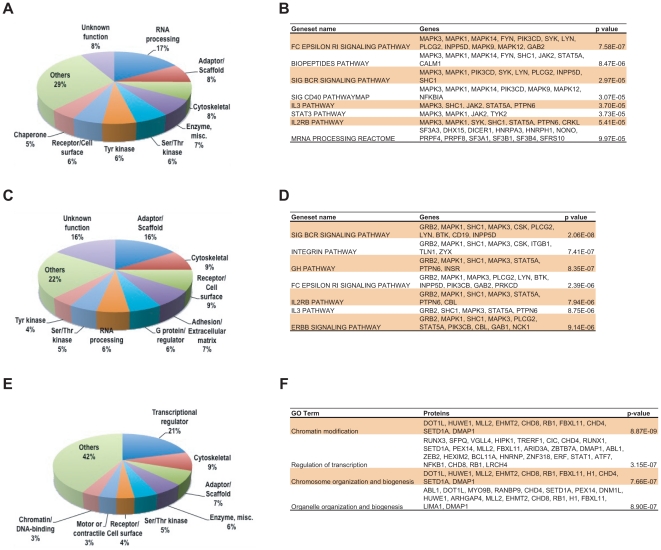
Classes of tyrosine and serine/threonine phosphorylated proteins that are responsive to FLT3 inhibitor. (A) and (B) Classes of proteins in which phosphotyrosine sites were identified in response to FLT3 inhibitor in MV(4;11) and Molm 14 AML cell line,. Only the top 8 classes were shown, the rest were classified as other. GSEA analysis revealed top ranked pathways that are enriched in drug sensitive tyrosine phosphorylated proteins using curated gene sets from molecular signature database. (C) and (D) Classes of proteins in which phosphotyrosine sites were identified in response to FLT3 inhibitor in SEM cell line. GSEA analysis revealed top ranked pathways that are enriched in drug sensitive tyrosine phosphorylated proteins using curated gene sets from molecular signature database. (E) and (F) Classes of proteins in which PXSP/PXTP sites were identified in response to FLT3 inhibitor in MV(4;11) cell line. BiNGO Gene Ontology (GO) analysis reveals cellular processes or pathways enriched in drug sensitive serine/threonine phosphorylated proteins.

To find out a subset of phosphorylated proteins and their respective sites that are regulated by FLT3, we employed stable isotope labeling with amino acids in cell culture (SILAC) to differentially label proteins derived from FLT3 inhibited versus uninhibited leukemia cell lines. We used MLN518, a quinazoline-based inhibitor of the type III receptor tyrosine kinases FLT3, PDGFRA, CSF1R, and C-KIT[29]. MV(4;11), Molm 14, and SEM are sensitive to MLN518 (Figure S1A). K562, which is driven by BCR-ABL and insensitive to FLT3 inhibitors, was included as a negative control. To address the off target effects of MLN518, we checked the phosphorylation and expression of CSF1R, C-KIT, and PDGFRA by Western blot analysis (Figure S1B). Clearly, none of these kinases were phosphorylated and expressed in the cell lines tested. Thus, FLT3 appears to be the major target of MLN518 in MV(4;11), Molm 14, and SEM cells. We set a 2-fold change in phosphopeptide intensity as a threshold to define MLN518 sensitive site. From duplicate runs, we identified 184 sites within 152 proteins and 39 sites within 33 proteins sensitive to MLN518 in MV(4;11) and Molm 14 cells, respectively. Very few, if any, MLN518 sensitive sites were observed in K562 cells (Table S3). Besides FLT3 tyrosine phosphorylation, no other tyrosine kinases were greatly inhibited by the treatment of MLN518 in these cell lines. Thus, most MLN518 sensitive sites appear to be regulated by FLT3 directly or downstream to FLT3. Of the proteins regulated by FLT3 many fall within expected protein types, such as adaptor/scaffold, proteins involved in cytoskeletal organization, enzymes, serine/threonine kinases, and tyrosine kinases (Figure 2A). Others are within protein types not previously associated with FLT3 signaling such as RNA processing, cell surface proteins, and chaperones. Notably, 27 of 28 proteins involving RNA processing identified in MV(4;11) cells were regulated by FLT3 inhibitor, which may reflect a cell type specific event. To further dissect signaling pathways regulated by FLT3-ITD, we applied Gene Set Enrichment Analysis (GSEA) [30] and identified Fc epsilon RI-mediated signaling pathways, B cell receptor signaling, CD40, IL2/3, and STAT3 pathways were enriched in FLT3-ITD regulated phosphoproteins, some of which are known to be involved in the pathogenesis of AML (Figure 2B).

**Figure 3:**
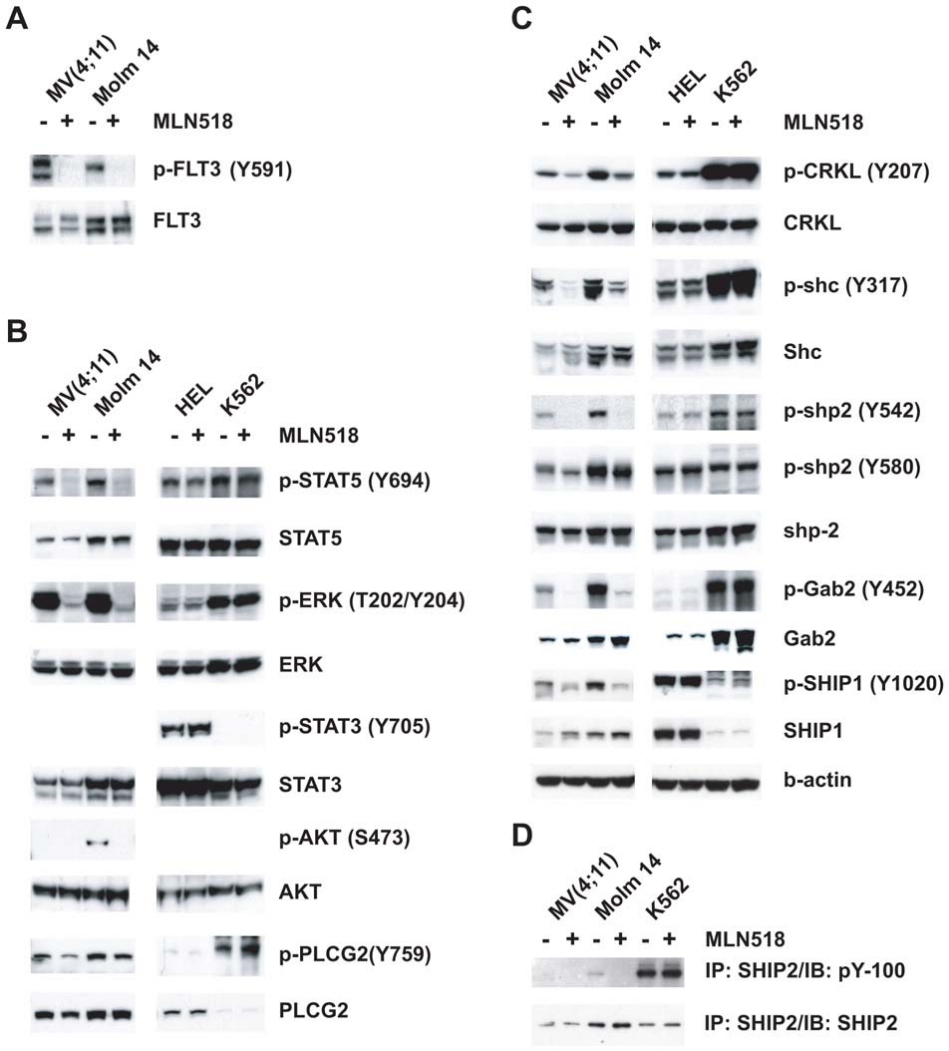
Western blot analysis confirms FLT3 inhibitor responsive pTyr-sites in AML cell lines. (A), (B), (C), and (D) Immunoblotting of MV(4,11) and Molm 14 cells treated with 2 uM FLT3 inhibitor (MLN518) for 2 hours with different phospho-specific antibodies. HEL and K562 cells were included as controls.

Some of the known FLT3 regulated phosphorylation proteins were significantly inhibited by MLN518, including FLT3 itself, and its downstream effectors, SHC, STAT5A, and ERK. In addition, we identified phosphorylation sites on adaptor proteins previous linked to wild type FLT3 signaling, such as GAB2, SHP-2, PLCG2, and SHIP1 [31], [32], [33], [34], [35]. Besides SHIP1, a more ubiquitously expressed phosphatase (SHIP2) is also downregulated by MLN518. Y986 on SHIP2 lies within a NPXY motif, which may interact with SHC in response to FLT3 signaling. In addition, quite a few phosphorylation sites on SRC family kinases were sensitive to MLN518, such as HCK, LYN, LCK, FYN, and FGR. This observation is consistent with the proposed role of SRC family kinases, especially LYN, in FLT3 signaling [36]. Some of these SILAC-MS results were further confirmed by Western blot analysis (Figure 3).

**Figure 4:**
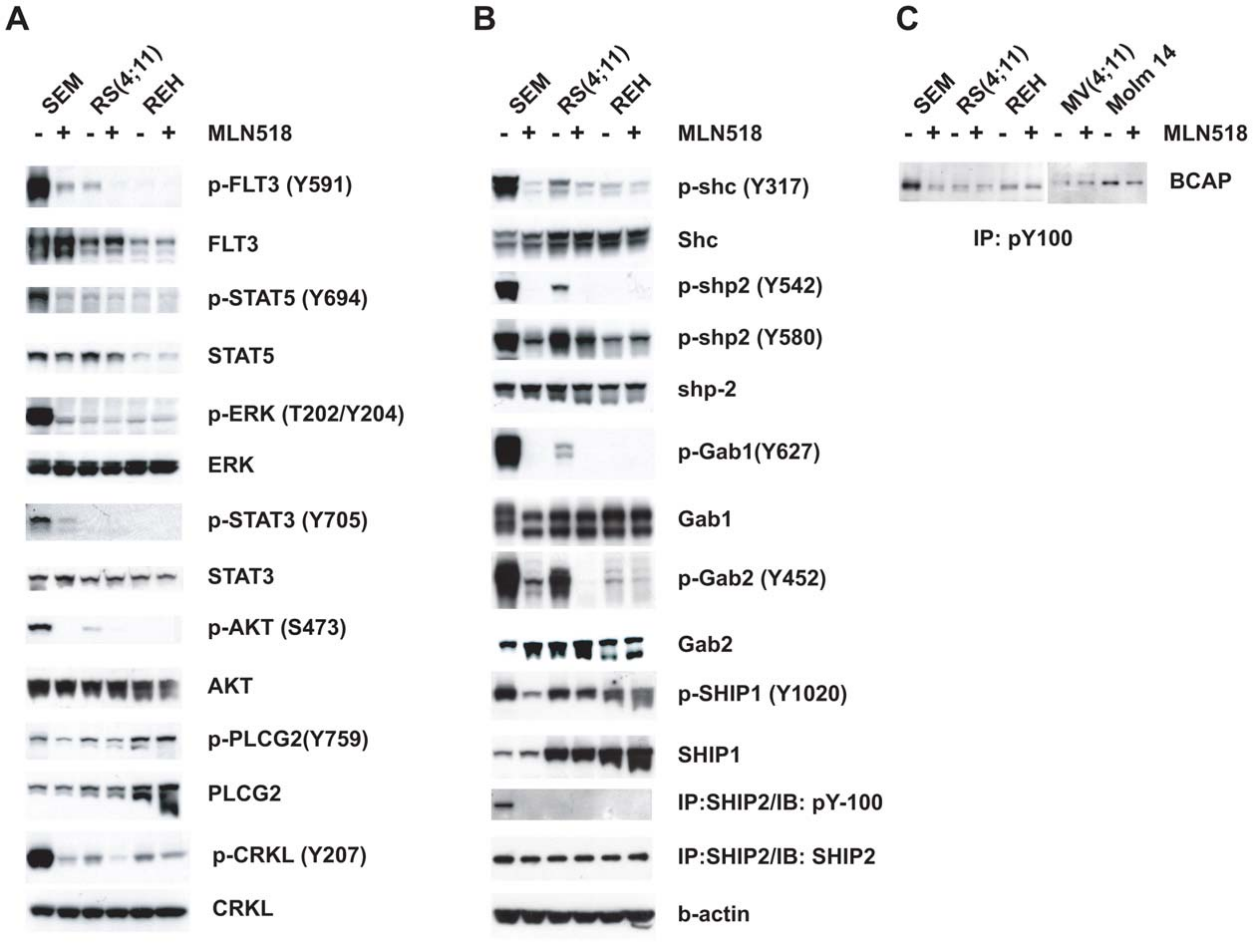
Western blot analysis confirms FLT3 inhibitor responsive pTyr-sites in B-ALL cell lines. (A), (B), and (C) Immunoblotting of SEM cells treated with 2 uM FLT3 inhibitor for 2 hours with different phospho-specific antibodies. RS(4,11) and REH cells were included as controls.

In addition to FIT3-ITD AML cell lines, we identified a total of 160 MLN518 sensitive sites from 120 proteins in SEM cells, significantly extended our knowledge of FLT3 signaling in ALL. Adaptor/scaffold, cytoskeletal proteins, cell surface molecules, and adhesion/extracellular matrix proteins make up the majority of protein types (Figure 2C). Similar to FLT3-ITD cell lines, B cell receptor signaling, integrin, growth hormone, and Fc epsilon RI-mediated signaling pathways were highlighted (Figure 2D). Besides Y842, two additional sites on FLT3 (Y630 and Y726) were inhibited by MLN518. Moreover, tyrosine phosphorylation of several components of B cell receptor (BCR) signaling pathways was also inhibited, including CD19, LAB, BCAP, LYN, BTK, GRB2, and GAB1/2/3. We confirmed a subset of phosphorylation sites by Western blot using phosphospecific antibodies, including BCAP, GAB1, and GAB2 (Figure 4). These results suggest that instead of activating novel signaling pathways, FLT3 utilizes components of BCR signaling pathway to promote proliferation and survival of lymphoid cells in B cells acute lymphoblastic leukemia. Some of the tyrosine phosphorylation sites regulated by MLN518 were further confirmed by Western blot with another FLT3 inhibitor, Sorafenib (Figure S2).

### Identification of MLN518-responsive serine and threonine phosphorylation sites

Because FLT3 receptor signaling engages the MAPK pathway and also regulated the cell cycle [37], we applied the same immuno-affinity profiling strategy, but using instead of a phospho-tyrosine antibody, two phospho-specific antibodies that recognize the PXSP and PXTP motifs, which are common substrate recognition motifs of MAPKs and CDKs. In brief, SILAC supernatants (after pY-100 immunoprecipitation) from MV(4;11), SEM, and K562 cells were sequentially immunoprecipitated with PXSP and PXTP motif antibodies, and analyzed by LC-MS/MS mass spectrometry. In MV(4;11) cells, we identified 150 sites from 88 proteins that are sensitive to MLN518 (Figure 2E and Table S3), 21% of them are from transcription factors. Consistent with this finding, gene ontology (GO) analysis revealed that biological processes, such as chromatin modification and regulation of transcription, were significantly enriched (Figure 2F). Among FLT3 regulated serine sites, phosphorylation of p70S6K^Ser424^ was further confirmed by Western blot with phospho-specific antibody (Figure S1C), locating this growth promoting kinase downstream to FLT3 in leukemic cells. In addition, FLT3 regulates phosphorylation of NFkB-p105^Ser907^, a precursor of the NFkB-p50 subunit. Dephosphorylation of NFkB-p105^Ser907^ accelerates the processing of p105 to p50 [38], [39]. ERF^Thr526^ is one of the sites most inhibited by MLN518 in MV(4;11). ERF, an ets domain transcriptional repressor, serves as a link between RAS/MAPK pathway and cell cycle progression [40]. Phosphorylation of ERF^Thr526^ by ERK renders it unable to repress *c-myc* and *cdc2* expression [41]. Thus, ERF maybe a target for inactivation by FLT3, possibly linking FLT3 with c-myc de-regulation in AML. FLT3 also regulates phosphorylation of both histone methyltransferase (MLL2) and histone demethylase (FBXL11), suggesting that FLT3 may play a role in epigenetic regulation. It is interesting to note that FLT3 activation leads to phosphorylation of AML1 on both serine (249 and 276) and threonine sites (273), possibly through an ERK dependent mechanism [42]. While we identified many MLN518 sensitive tyrosine sites in SEM cells, we observed very few PXSP/PXTP sites in SEM cells, including ERF, CapZIP, and AML1 (Table S3).

### Global phosphorylation profiling of bone marrow cells from AML patient samples

**Figure 5:**
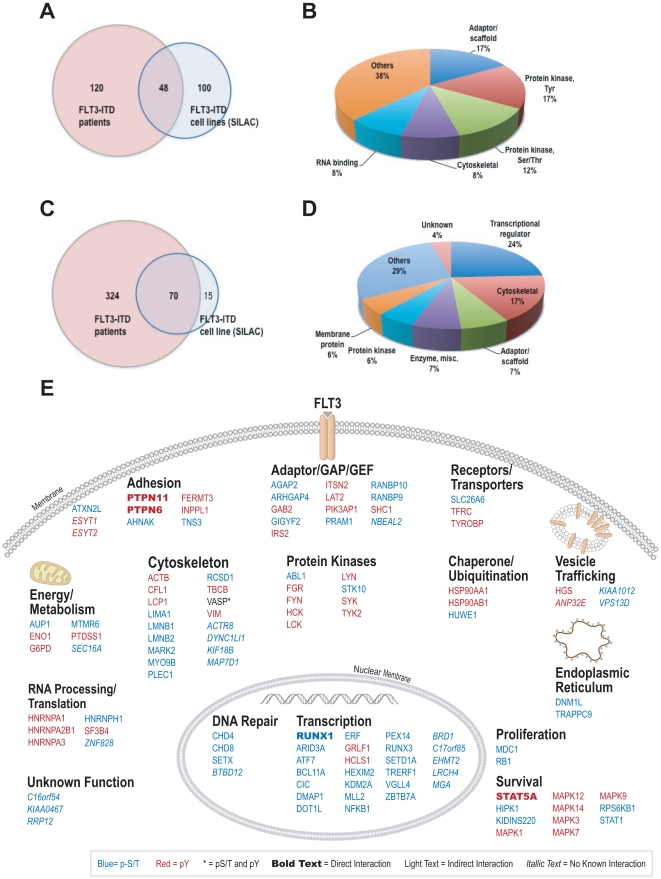
Presence of FLT3 regulated phosphoproteins in FLT3-ITD primary AML patients. (A) and (B) Venn diagram showed that MLN518 sensitive tyrosine phosphorylated proteins (> = 2 fold) identified in FIT3-ITD cell lines were also present in FLT3-ITD primary AML patients. Pie chart showed the protein types of these overlapped proteins. (C) and (D) Venn diagram showed that MLN518 sensitive serine/threonine phosphorylated proteins (> = 2 fold) identified in FIT3-ITD cell lines were also present in FLT3-ITD primary AML primary patients. Pie chart showed the protein types of these overlapped proteins. (E) Regulatory networks of FLT3 signaling that have in vivo relevance. Phosphoproteins in the network were derived from proteins in the overlapped region in figure 5A and 5C.

To understand the role of FLT3 activation in primary patients, we profiled bone marrow cells from AML samples with known FLT3 mutational status. We identified 209 tyrosine phosphorylation sites from over 200 proteins in 6 AML patients, three of which contains FLT3-ITD mutation (Table S1 and S2). We detected FLT3 tyrosine phosphorylation at its activation loop (Y842) in all three FLT3-ITD patients (Table S2). Of note, tyrosine phosphorylation of STAT5, a major downstream target of FLT3, was only present in FLT3-ITD patients. Phosphorylation of FLT3 was also detected in one wild type FLT3 case, consistent with the observation that FLT3 is expressed in a high percentage of AML patients. SRC family kinases, LYN and LCK, were abundantly phosphorylated on their activation sites in these AML samples, consistent with the importance of activation of SRC family kinases in AML survival [43]. 48 MLN518 sensitive tyrosine phosphorylated proteins from adaptor/scaffold, tyrosine kinases, serine/threonine kinases, and other protein types, found in FLT3-ITD AML cell lines are also present in these FLT3-ITD patients (Figure 5A and 5B, Table S4), suggesting that these proteins might play important roles of FLT3-ITD signaling in vivo.

Besides phosphotyrosine profiling, we performed IAP-MS analysis with PXSP and PXTP motif antibodies on these AML patients, and identified 859 serine/threonine sites from 486 proteins (Table S1 and S2). Many MLN518 sensitive pSer/Thr sites identified in MV(4;11) cell line are also present in FLT3-ITD AML patients, such as p70S6K^Ser424^, NFkB-p105^Ser907^, AML^Ser249,276,Thr273^, and ERF^Thr526^. Overall, we identified 70 drug sensitive serine/threonine phosphorylated proteins (transcription factors, cytoskeletal proteins, adaptors, and others) in 3 FLT3-ITD patients (Figure 5C and 5D, Table S4). Overall, we identified 117 FLT3 regulated tyrosine and serine/threonine phosphorylation proteins in 3 FLT3-ITD leukemia patients. They are from different cellular compartments and involved in diverse cellular processes, only a few of them have direction interactions with FLT3 (Figure 5E). Thus, this study expanded our understanding of FLT3 signaling in vivo.

## Discussion

Deregulated tyrosine kinases have been frequently implicated in the pathogenesis of cancer, including AML [44]. However, tyrosine phosphorylation represents less than 2% of total protein phosphorylation [45], [46], making the study of phosphorylated tyrosine residue challenging. For the past decade, our knowledge of FLT3 signaling pathways has been painstakingly accumulated, mainly through the study of individual molecules in certain pathways. In our study, using PhosphoScan®, a phosphotyrosine profiling proteomics approach we profiled 11 leukemia cell lines (8 AML and 3 B-cell ALL), and six primary AML patients. We identified over 1000 tyrosine phosphorylated sites from about 800 proteins. Our study significantly expands the inventory of phosphorylated proteins associated with FLT3 signaling in human AML and B-ALL. Our data thus provide a unique insight into in vivo FLT3 signaling components and their relevant regulatory sites. This global, unbiased view of the in vivo phosphoproteome revealed a large number of cellular proteins phosphorylated, and identified an important subset of proteins and their phosphorylation sites regulated in response to FLT3 inhibitor.

Recent Studies done by Choudhary et al. and Zhang et al., which represent the first two large-scale Phospho-profiling of mutant FLT3 signaling, provide broad view of oncogenic FLT3 signaling to date. However, these studies were performed with overexpression of mutant FLT3 in murine systems. In contrast to the study done by Choudhary et al., which was performed with PKC412 for 12 hours, our study was done with MLN518 for 2 hours, which reflects early changes in protein phosphorylation. We observed FLT3 regulates tyrosine phosphorylation in protein kinases, adaptor/scaffold proteins, and phosphatase. In addition, some FLT3 regulated sites are tied to many previously unknown cellular systems, as demonstrated here by identification of regulated sites on cell surface proteins, cytoskeletal proteins, RNA processing proteins and other important protein classes. The diversity of protein classes found to be regulated by FLT3 inhibitor suggests that FLT3 affects a broad spectrum of signaling pathways and cell functions not limited to proliferation and survival. For example, Y399 of DNMT1 is a drug sensitive site, raising the possibility that that FLT3-ITD regulates the activity of DNMT1, and affects global DNA methylation patterns in FLT3-ITD leukemic cells. Not only does FLT3 activate tyrosine signaling pathways, but it also regulates epigenetic events through phosphorylation of transcription factors on serine/threonine residues. Future studies should be aimed at connecting these data directly to downstream gene-expression changes as measured by microarray technology.

Some of the FLT3 regulated proteins contained more than one regulated site, such as Y67, 88, and 139 of cofilin1 in MV(4;11) cells, as well as Y266 and 476 of GAB2 in Molm 14 cells. Our data also showed that phosphorylation of different sites within the same protein can also be differentially regulated. For example, Y383 and Y598, but not Y208 on GIT1 (G protein-coupled receptor kinase interactor 1) are responsive to MLN518 In MV(4;11). A similar observation was made for tyrosine sites on ENO1 and HCK. This highlights the importance of measuring the degree of site-specific phosphorylation rather than phosphorylation the protein as a whole in order to obtain an accurate picture of a protein's function.

In SEM cells, we observed multiple FLT3 regulated sites on key elements of B-cell receptor signaling pathways such as BCAP, CD19, LAB, DOK1, GAB1, and GAB2. In addition, we confirmed phosphorylation of BCAP is regulated by FLT3. BCAP binds PI3K and plays an essential role in B cell development [26], [28]. However, immunoprecipitation with FLT3 antibody did not pull down CD19, BCAP and LAB (data not shown). Thus, FLT3 might indirectly regulate tyrosine phosphorylation of these proteins. Since B cell receptor signaling is involved in B cell development, activation and differentiation [25], oncogenic FLT3 might provides proliferative and survival signal for B cells by regulating components of B cell receptor signaling. It is plausible that oncogenic FLT3 may cooperate with other oncogenic events such as MLL fusions to promote leukemogenesis of B-ALL [47].

In this study, we identified five FLT3 tyrosine phosphorylation sites in primary leukemia cell lines, adding new information on how phosphorylation may affect the activity of wild-type FLT3 and FLT3-ITD. FLT3 has been implicated in the pathogenesis of both AML and B-ALL. We observed that in both AML and B-ALL cell lines, FLT3 activates many of the similar signaling pathways, such as JAK-STAT pathway, RAS-MAPK pathway, and phospholipase C-gamma (Figure 3 and 4). Yet, a key difference is the apparent recruitment of the BCR complex by FLT3 in B-ALL. Our data is therefore consistent with the observation that FLT3 inhibitors have therapeutic effects in B cell acute lymphoblastic leukemia with high level of FLT3 expression [8]. This suggests that FLT3 pathogenesis in B-ALL could depend on elements of that pathway which could be additionally targeted by tailored therapies.

When we compared phosphotyrosine profile between two FLT3-ITD cell lines (MV(4;11) and Molm 14) and three FLT3-ITD AML patients, we noticed that 77% (130 out of 168) of phosphoproteins identified in AML patients were also present in these two cell lines, suggesting that FLT3-ITD cell lines are good models to understand FLT3-ITD signaling for primary patients (Figure S3). On the other hand, there are a large number of phosphoproteins identified in FLT3-ITD cell lines not present in primary patients. It could be due to the lengthy period required to collect bone marrow and lyse red blood cells, during which a significant amount of tyrosine phosphorylation events were lost. Nonetheless, tyrosine phosphorylated proteins, like FLT3, ESYT1/2 (FAM62A/B), HGS, HSP90AA, INPPL1(SHIP-2), LYN, SF3B4, SHC1, STAT5A, and VASP, are not only enriched in Molm14 and MV(4;11) cell lines with statistical significance (FDR<0.05) and regulated by FLT3 inhibitor, but also present in FLT3-ITD AML patients. While some of these proteins are known components of FLT3 signaling pathways, the function of other proteins remain to be determined.

Several small molecule FLT3 inhibitors are in phase I/II clinical trials. However, the fundamental relationship between FLT3 inhibition, its consequences on downstream signaling, and the onset of cytotoxicity remains poorly understood [13]. A recent study showed that cytotoxic responses to CEP-701 and PKC412 were highly heterogeneous and were only weakly associated with FLT3 expression level and mutation status [48]. Clearly, if FLT3 small molecule inhibitors were to have an impact on AML treatment, a better understanding of how FLT3 signaling contributes to the disease, and how FLT3 drugs work at the cellular level, would be necessary. This study not only provides a comprehensive view of FLT3 regulated signaling pathways in human AML cell lines, but also gives us an initial glance onto the activation of these pathways in primary AML patient samples. We showed that oncogenic FLT3 affects multiple signaling pathways in human AML patients and regulates proteins from different cellular compartment and diverse cellular processes. Targeting pathways of Fc epsilon RI-mediated signaling, MAPK, BCR, and CD40 signaling may offer new hope to treat FLT3-ITD AML. This study opens the door to a deeper understanding of FLT3 signaling networks, to identify novel and better biomarkers to trace clinical response to FLT3 inhibitors. In addition, this study provides a valuable resource for the scientific community to further investigate oncogenic FLT3 signaling in primary AML patients.

## Materials and Methods

### Cell culture and AML patient samples

8 human AML cell lines (MV(4;11), Molm 14, Marimo, Me-F2, KY821, OCI/AML3, Nomo-1, and ML-1) and 3 human B-ALL cell lines (SEM, RS(4;11), and REH) were included in mass spectrometry analysis. Both MV(4;11) and Molm 14 contain FLT3-ITD mutation. SEM contains amplified copy number of FLT3. Detailed history about these cell lines were provided in table S1. K562 cells were obtained from American Type Culture Collection (ATCC). OCI/AML3, Nomo-1, GDM-1, HEL, M-07e, SEM, RS(4;11), and REH cells were obtained from the German National Resource Centre for Biological Material (DSMZ). M-07e was grown in 80%IMDM with 20%FBS, plus 10 ng/ml GM-CSF. All other cell lines were grown in RPMI-1640 with 10% FBS. Cells were treated for 2 hours (immunoblotting and SILAC) with 2 uM MLN518 (Tandutinib, a FLT3 inhibitor from Millennium Pharmaceuticals, Inc., The Takeda oncology company) before lysis. For dose response curves, cells were incubated for 48 hours in the presence of different concentration of MLN518, and the number of viable cells was determined with the CellTiter 96 AQ_ueous_ One solution cell proliferation assay (Promega). Collection and use of de novo AML patient bone marrow samples were approved by the human subjects Institutional Review Boards of Oregon Health & Science University and Portland VA Medical Center with written consent from patients. Patient information was not revealed in this study, and the data were analyzed anonymously.

### Phosphopeptide immunoprecipitation and analysis by LC-MS/MS Mass Spectrometry

Phosphopeptides were prepared using PhosphoScan® Kit (Cell Signaling Technology). Briefly, about 2×10^8^ cells (or 1×10^8^ bone marrow cells) were lysed in urea buffer, trypsin digested lysate were purified by Sep-pak C_18_ column. Then, lyophilized peptides were redissolved and immunoaffinity purified with pY-100 antibody, which interacts with a broad range of tyrosine-phosphorylated peptides. pTyr-containing peptides were concentrated on reverse-phase micro tips. Supernatants after pY-100 immunoprecipitation were further immunoaffinity purified with PXSP and PXTP (Phospho-MAPK/CDK substrate) motif antibodies, which detect phospho-serine in a PXS*P or phospho-threonine in a PXT*P motif. Tandem mass spectra (ms/ms) were collected in a data-dependent fashion with an LTQ ion trap mass spectrometer or and LTQ-Orbitrap mass spectrometer (ThermoFinnigan) in which the top 10 ions were selected for ms/ms. Sequest (Thermo Fisher Scientific) searches were done against the NCBI human database released on July 02, 2009, (containing 37,391 proteins), allowing for phosphorylation (STY+80) and oxidized methionine (M+16) as differential modifications using a precursor tolerance of 1.2 Da for LTQ data or 50 ppm LTQ Orbitrap data. LTQ data was searched fully tryptic, while Orbitrap data was searched with partial tryptic specificity. The PeptideProphet probability threshold was chosen to give a false positive rate of 1% for the peptide identifications (Table S1) [49]. Phosphorylation sites were localized using the Ascore[50]. Data from duplicate experiments were combined.

### SILAC analysis of MLN518-treated cells

MV(4;11), Molm 14, SEM, and K562 cells were grown in RPMI-1640 (lacking arginine and lysine) supplemented with 10% dialyzed fetal bovine serum, and L-lysine/HCl and L-arginine/HCl (Sigma, St Louis, MO) for light cultures or L-arginine/HCl (U-13C6, 98%) and L-lysine:2 HCl (U-13C6, 98%; U-15N2, 98%) (Cambridge Isotope Laboratories, Andover, MA) for heavy cultures, as previously described [51], [52], [53]. After lysis, 1×10^8^ cells from either heavy or light cultures were combined and carried through the phosphopeptide immunoprecipitation protocol. Methods for LTQ-FT MS, Sequest searches and Vista (pTyr, pSer, and pThr SILAC samples) were described previously [54], [55].

### Statistical and computational analysis

We used the following statistical and computational tools from Gene Set Enrichment Analysis (GSEA, Broad Institute of MIT and Harvard) for Molecular Signature Database analysis; BIOBASE Knowledge Library for Gene Ontology analysis. STRING 8.2 for potential protein protein interaction analysis [56].

### Western blotting

Cells were lysed in 1× cell lysis buffer (Cell Signaling Technology) supplemented with Protease Arrest™ (G Biosciences) and separated by electrophoresis. All antibodies and reagents for immunoblotting were from Cell Signaling Technology, Inc.

## Supporting Information

Figure S1 **FLT3-ITD cell lines are sensitive to its inhibitor.** (**A**) Sensitivity of AML and B-ALL cell lines to FLT3 inhibitor (MLN518). % control means percent of viable cells after drug treatment as compared to untreated control. (**B**) Phosphorylation and expression of CSF1R, C-KIT, and PDGFRA in leukemia cell lines. GDM-1, M-07e, and H1703 cell lines are positive controls for the expression of CSF1R, C-KIT, and PDGFRA, respectively. (**C**) Phosphorylation of p70S6 kinase is inhibited by FLT3 inhibitor.
(TIF)

Figure S2 **Western blot analysis confirms MLN518 responsive pTyr-sites in AML and B-ALL cell lines by Sorafenib treatment.** (A), (B), and (C) Immunoblotting of MV(4,11), Molm 14, and SEM cells treated with 100 nM FLT3 inhibitor (sorafenib) for 2 hours with different phospho-specific antibodies. K562 cells were included as controls.
(TIF)

Figure S3 Venn diagram showed the overlap of tyrosine phosphorylated proteins identified between two FLT3-ITD AML cell lines and three FLT3-ITD primary AML patients.
(TIF)

Table S1 Phosphopeptides identified by LC-MS/MS in AML and B-ALL cell lines, as well as primary AML patients. P1–P3 are FLT3 wild type AML patients, P4–P6 are FLT3-ITD AML patients. ‘Y*’, ‘S*’/, and ‘T*’ in peptide indicates phosphorylated tyrosine, serine, or threonine residue, respectively. Spectra links were provided, as well as other important mass spectrometry parameters (XCorr, Ascore, etc.). False discovery rate (fdr) for each dataset was also listed.
(XLS)

Table S2 Non-redundant phosphopeptides identified by LC-MS/MS in this study presented in a format that is based on individual cell line or AML patient. ‘y’, ‘s’, and ‘t’ in peptide indicates phosphorylated tyrosine, serine, or threonine residue, respectively. Data from duplicate experiments were combined. Tyrosine kinases profile in AML cell lines and patients were summarized as well. ‘§’ indicates known phosphorylation site.
(XLS)

Table S3 Phosphotyrosine SILAC data of MV(4,11), Molm 14, SEM, and K562 cell lines, and phosphoserine (PXSP) and phosphothreonine (PXTP) SILAC data of MV(4,11), SEM, and K562 cell lines, treated with FLT3 inhibitor (MLN518). ‘Y*’ ‘s’, and ‘t’ in peptide indicates phosphorylated tyrosine, serine, or threonine residue, respectively. ‘§’ indicates known phosphorylation. Results from duplicate run were combined.
(XLS)

Table S4 List of proteins and their protein types identified in SILAC study of FLT3-ITD cell lines (> = 2 fold) that were also present in three FIT3-ITD primary patients. Both tyrosine phosphorylated and serine/threonine phosphorylated proteins were listed.
(XLS)
