# Supplementary figures and images for: Survey of Activated FLT3 Signaling in Leukemia
Source: PLoS One. 2011 Apr 28;6(4):e19169. doi: 10.1371/journal.pone.0019169 (PMC3084268; doi:10.1371/journal.pone.0019169)

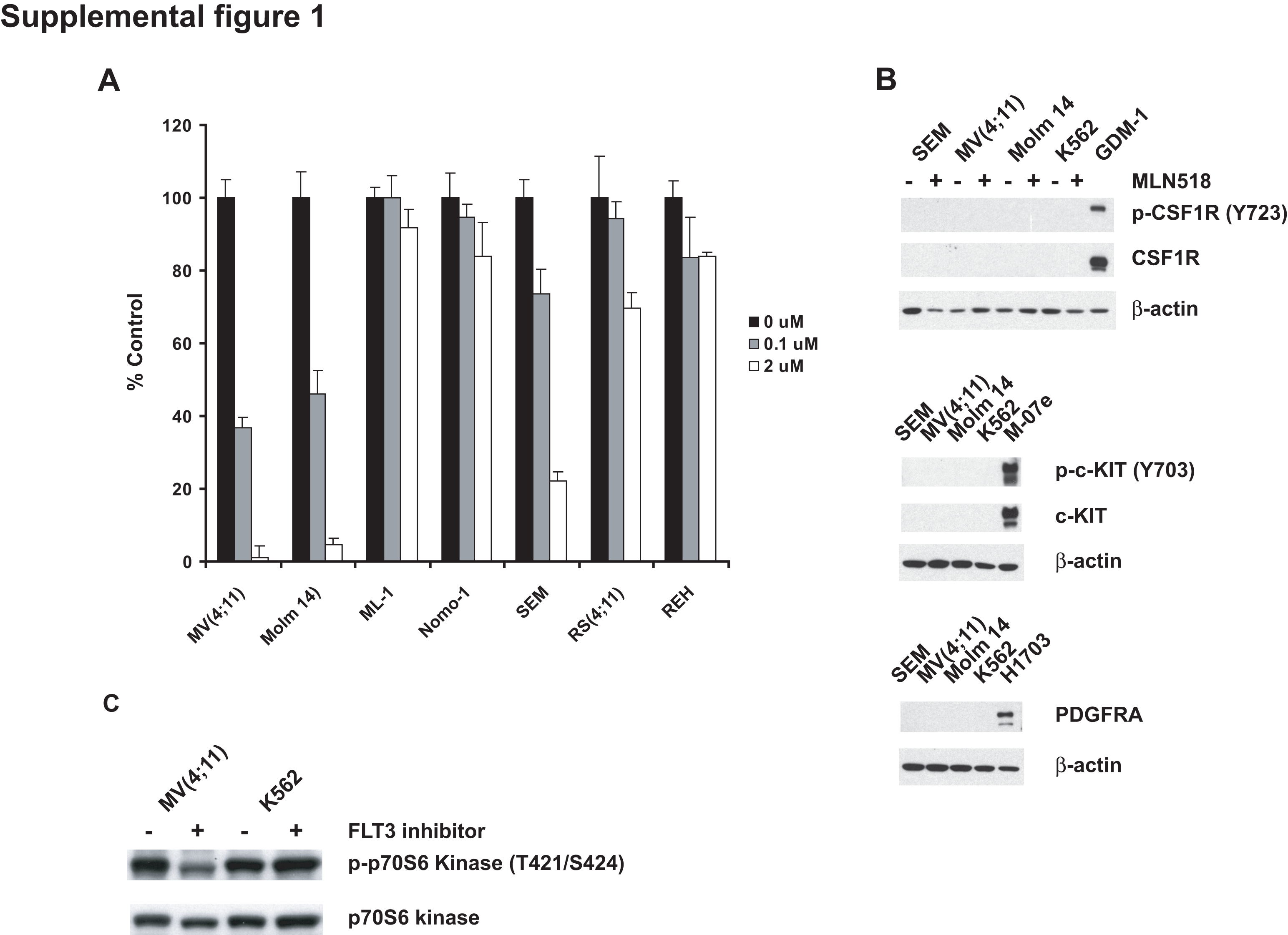

Supplement: Figure S1 — FLT3-ITD cell lines are sensitive to its inhibitor. (A) Sensitivity of AML and B-ALL cell lines to FLT3 inhibitor (MLN518). % control means percent of viable cells after drug treatment as compared to untreated control. (B) Phosphorylation and expression of CSF1R, C-KIT, and PDGFRA in leukemia cell lines. GDM-1, M-07e, and H1703 cell lines are positive controls for the expression of CSF1R, C-KIT, and PDGFRA, respectively. (C) Phosphorylation of p70S6 kinase is inhibited by FLT3 inhibitor. (TIF) [file pone.0019169.s001.tif]

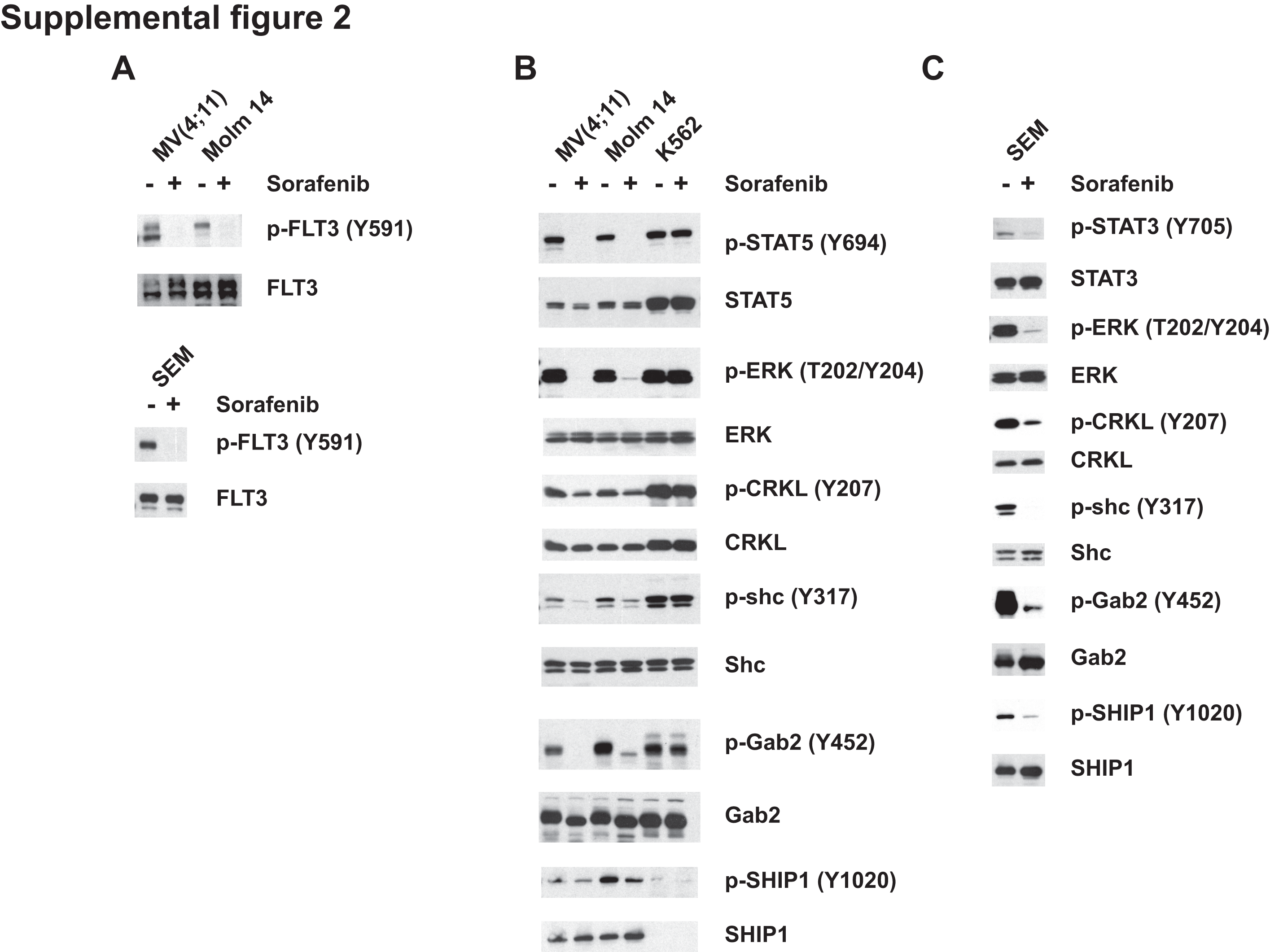

Supplement: Figure S2 — Western blot analysis confirms MLN518 responsive pTyr-sites in AML and B-ALL cell lines by Sorafenib treatment. (A), (B), and (C) Immunoblotting of MV(4,11), Molm 14, and SEM cells treated with 100 nM FLT3 inhibitor (sorafenib) for 2 hours with different phospho-specific antibodies. K562 cells were included as controls. (TIF) [file pone.0019169.s002.tif]

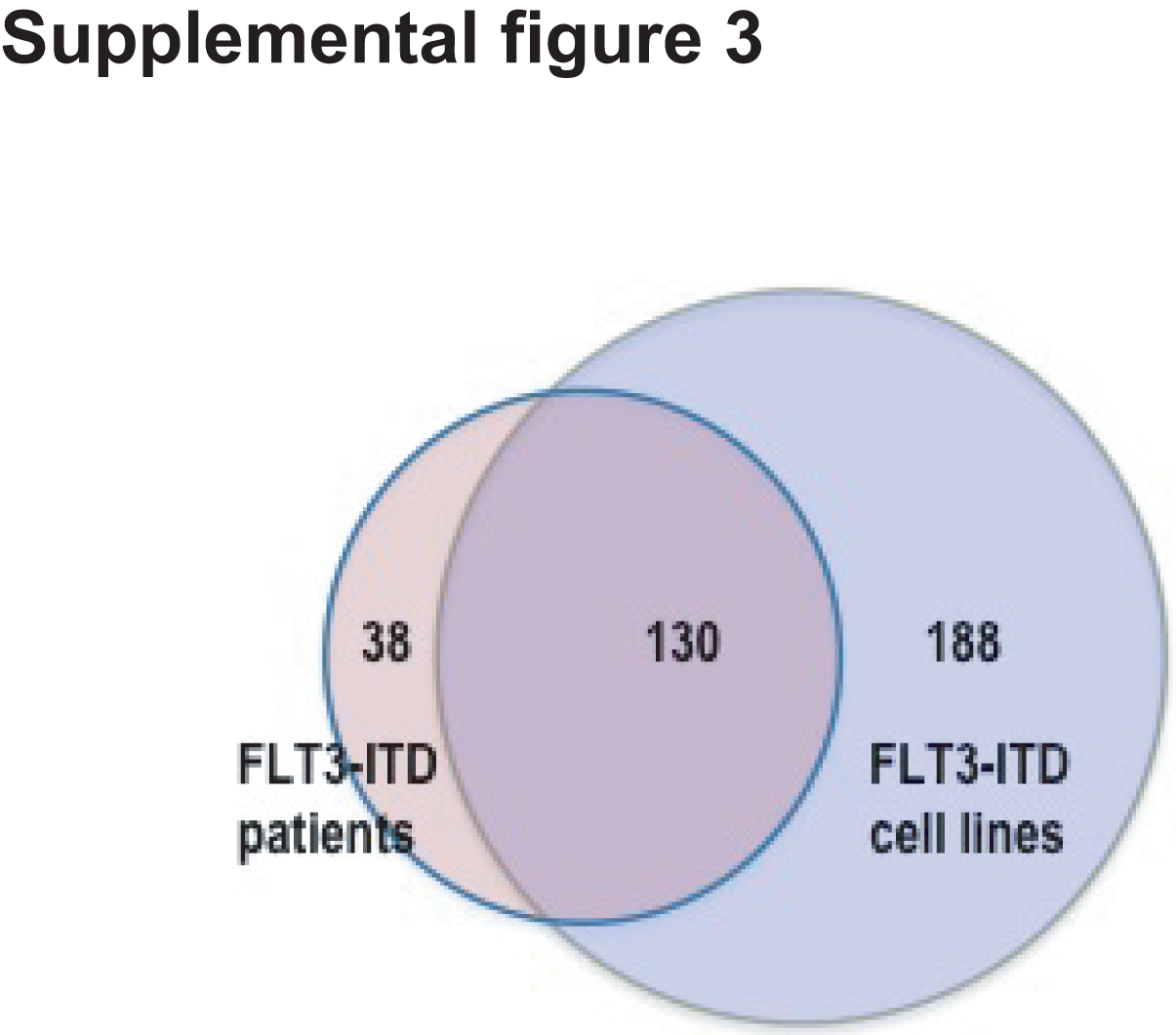

Supplement: Figure S3 — Venn diagram showed the overlap of tyrosine phosphorylated proteins identified between two FLT3-ITD AML cell lines and three FLT3-ITD primary AML patients. (TIF) [file pone.0019169.s003.tif]
